# A Method for Assigning Priorities to United States Measurement System (USMS) Needs: Nano-Electrotechnologies

**DOI:** 10.6028/jres.114.017

**Published:** 2009-08-01

**Authors:** Herbert S. Bennett, Howard Andres, Joan Pellegrino

**Affiliations:** National Institute of Standards and Technology, Gaithersburg, MD 20899; Energetics Incorporated, Columbia, MD 21046

**Keywords:** Borda count method, measurement needs, median method, nano-electrotechnologies, priorities, rankings, standards, statistical significance, United States Measurement System

## Abstract

In 2006, the National Institute of Standards and Technology conducted an assessment of the U.S. measurement system (USMS), which encompasses all private and public organizations that develop, supply, use, or ensure the validity of measurement results. As part of that assessment, NIST collaborated with Energetics Incorporated to identify and authenticate 723 measurement needs that are barriers to technological innovations. A number of these measurement needs (64) are relevant to accelerating innovation and commercialization of nano-electrotechnologies. In this paper, we apply the taxonomy from a 2008 international survey that established a global consensus of priorities for standards and measurements in nano-electrotechnologies to rank in priority order the relevant 64 USMS-identified measurement needs. This paper presents a method for assigning priorities that is statistically based and represents a global consensus of stakeholders. Such a method is needed because limited resources exist to address the large number of measurement needs in nano-electrotechnologies, and the most critical measurement needs should be addressed first.

## 1. Introduction

Nano-electrotechnologies are expected to be among the key technologies of the 21st century. They have enormous potential for the development of new products with exceptional performance. Nano-electrotechnologies will enable society to take advantage of economic successes, as well as improvements in the quality of life by using nano-enabled products. A strong measurements and standards infrastructure is essential if the investments in nanotechnologies are to be successful for the delivery of useful products and services. [1,2] International commerce in nano-electrotechnologies will require technically valid standards and related measurements that are suitable for use in any nation. These standards must therefore be developed with input from all stakeholders.

According to a recently published report of Semiconductor Equipment and Materials International (SEMI) in cooperation with the Semiconductor Industry Association (SIA) [3] and by the RNCOS Group [4], the materials and equipment market for nanoelectronics was US$ 1.8 billion in 2005 and is expected to be US$ 4.2 billion in 2010. The continued rapid growth of this and other nano-electrotechnologies-based industries has required increased international standardization activities to support equitable and efficient business models.

Applications of nano-electrotechnologies include: [5]

- Analytical equipment and techniques for measurement of electrotechnical properties
- Fabrication tools for integrated circuits (electronic, photonics, and optoelectronic)
- Nano-structured sensors
- Nano-electronics, materials and devices
- Optoelectronics
- Optical materials and devices
- Organic (opto) electronics
- Magnetic materials and devices
- Radio frequency devices, components, and systems
- Electrodes with nano-structured surfaces
- Electrotechnical properties of nanotubes/nanowires
- Fuel cells
- Energy storage devices (e.g., batteries)
- Bioelectronic applications
- Nano-enabled solar cells

Due to the large number of potential applications for nano-electrotechnologies and to the limited resources for development of standards, there is a need to prioritize future standardization work and make certain that the most important standards are developed first. A recent international effort in this regard is the NIST-Energetics-International Electrotechnical Commission (IEC) Technical Committee (TC) 113 International Survey. [6] The analysis described in this paper builds on this previous effort to prioritize standards and their associated measurement needs (MNs) by applying a method for assigning priorities that is statistically based and represents a global consensus of stakeholders.

### 1.1 Objectives

The objective of this paper is to use the results from previous efforts and analyses to demonstrate as a proof-of-concept a method for assigning priorities to several action items, which in this paper are USMS MNs. Section 2 summarizes the background, origin, structure, methodology, demographics, and results of prior efforts to prioritize measurement needs, including a 2006 assessment of the U.S. measurement system [7] and the IEC international Survey. [6] Section 3 contains the procedures by which we assign priorities to the 64 USMS MNs on nano-electrotechnologies identified in the 2006 assessment based on the Survey taxonomy. Section 4 contains a summary of the major results. Finally, Appendix A contains a listing of the 64 case studies of USMS MNs on nano-electrotechnologies.

Definitions:

*Nanotechnology* is the understanding and control of matter at dimensions between approximately 1 and 100 nanometers, where unique phenomena enable novel applications. Encompassing nanoscale science, engineering, and technology, nanotechnology involves imaging, measuring, modeling, and manipulating matter at this length scale. Dimensions between approximately 1 and 100 nanometers are known as the nanoscale. Unusual physical, chemical, and biological properties can emerge in materials at the nanoscale. These properties may differ in important ways from the properties of bulk materials and single atoms or molecules. [8]

*Nano-electrotechnologies* include the following areas at the nanoscale: nanostructured sensors; nano-electronics, nano-materials and nanodevices; optoelectronics; optical materials and devices; organic (opto)-electronics; magnetic materials and devices; radio frequency devices, components and systems; electrodes with nanostructured surfaces; electrotechnical properties of nanotubes/nanowires; analytical equipment and techniques for measurement of electrotechnical properties; patterning equipment and techniques; masks and lithography; performance, durability, and reliability assessment for nanoelectronics; batteries; fuel cells; and bioelectronic applications. [9]

## 2. Nanotechnology Measurement and Standards: Assessment and Prioritization

### 2.1 2006 Assessment of the U.S. Measurement System

As the national measurement institute for the United States, the National Institute of Standards and Technology (NIST) assists all stakeholders in selected fields with measurement and standards needs. The goal of NIST’s involvement is to enhance efficiency and productivity and to increase the rate of technological innovation. NIST is not a regulatory agency, but rather serves as a neutral third party often providing technical input in matters related to measurements and standards to a variety of customers such as standards committees, regulatory agencies, other government agencies, universities, and the private sector where appropriate. In 2006, NIST accepted the challenge to evaluate whether the U.S. measurement system (USMS) is meeting the nation’s measurement needs and produced an assessment detailing the findings. [7] Nanotechnology was one of the sector areas assessed in terms of measurement needs for accelerating technological innovation and commercialization.

As part of that assessment, NIST collaborated with Energetics Incorporated to identify and authenticate 723 measurement needs that are barriers to technological innovations. These measurement needs were derived from case studies and a review of technology roadmaps and reports in the public literature. Appendix B of the June 2006 USMS Assessment Report [7] contains 342 case studies of measurement needs (MNs), of which 64 cases studies concerned nano-electrotechnologies.

Based on their evaluation of measurement needs, the authors of the June 2006 USMS Assessment Report [7] made the following observations about nanotechnologies:

- Nanotechnology is unique among the sector/technology areas in its high demand for new advanced measurement instrumentation, which is needed to achieve accurate, high-resolution characterization of physical, chemical, and biological properties of materials at nanometer dimensions.
- Industry is limited not only in its ability to measure key parameters but also in its ability to identify which key parameters must be measured to meet anticipated regulations.
- The absence of measurement tools with the capability to measure properties of nanomaterials and nanodevices as they relate to functional performance and to make such measurements at speed are impediments to realization of nanotechnology products.
- Public-sector measurement providers that are linked to applied research sectors, production communities, market drivers, and user issues can help accelerate the innovative process.
- Innovative approaches to the measurement of nanoscale physical and chemical properties are key to technological innovation for nanotechnology, especially where the fundamental limits of current measurement techniques are being approached.

Industry technology roadmaps are an important source of measurement needs pertinent to nanotechnologies. These include the 2007 International Technology Roadmap for Semiconductors [1] and the 2003 Chemical Industry Roadmap for Nanomaterials by Design. [2] Prepared in conjunction with the USMS 2006 Assessment, the USMS Technology Roadmap Review Summary Report identified a number of measurement needs relevant to nanotechnologies, some of which are shown. [7]

- Sensing and detection devices operating at the nanoscale are likely to have myriad applications, including: detection of chemical, biological, radiological, and explosive elements; detection and treatment of infection, nutrient deficiency, and other health problems; tracking of food pathogens and agricultural products; nanoseparation and nanobioreactors; ensuring food safety; environmental monitoring; advanced protective clothing and filters and remediation of attacks; and creating anti-fouling nanosurfaces (e.g., packaging).
- A broad range of measurement needs associated with the understanding, characterizing, synthesizing, and manufacturing of new nanomaterials: characterization tools, methods, and instruments for properties measurement; tool development infrastructure; reference standards and protocols for synthesis and analysis protocols; robust measurement tools for manufacturing; characterization, measurement, and simulation probes for use during synthesis; and measurements for environmental, health, and safety impacts of nanomaterials.
- Measurement capabilities to further development and application of nanostructures as energy carriers (optimized energy transport); characterization methods, theory, imaging tools, and simulation and modeling to link nanoscale structure and function for nanomaterial assembly and architecture with the design of new materials for energy applications; and measurements to aid evaluation and development of carbon nanotubes for hydrogen storage.

### 2.2 NIST-Energetics-IEC TC 113 International Survey

In 2008, the IEC TC 113 on nano-electrotechnologies invited members of the international nanotechnologies community to respond to a Survey to identify those nano-electrotechnologies relevant to electronics and electrical products and systems for which standards are critically needed to accelerate innovation. The resulting Survey paper [6] contains the analyses of 459 Survey responses from 45 countries.

The Survey represents one way to begin building a consensus on a framework leading to nano-electrotechnologies standards development by standards organizations and national measurement institutes. The expectation was that responses to the Survey would enable the IEC TC 113 to:

- set procedures for ranking proposals and associated documents for new work in priority order;
- identify members for work groups on standards and associated documents; and
- make informed responses to proposals from IEC National Committees.

The distributions of priority rankings from all 459 respondents are such that there are perceived distinctions with statistical confidence between the relative international priorities for the several items ranked in each of the following five Survey category types:

1. Nano-Electrotechnology Properties,
2. Nano-Electrotechnology Taxonomy: Products,
3. Nano-Electrotechnology Taxonomy: Cross-Cutting Technologies,
4. IEC General Discipline Areas, and
5. Stages of the Economic Model.

Table 1 illustrates the category types and taxonomy employed in the Survey. We use *n_i_* in the next paragraph to denote the number of items listed in each category type. For example, *n_i_* equals 6 for category type Properties. One of the primary goals was to determine a consensus prioritization among the items listed for each of the category types. With this goal in mind, the Survey required the respondents to rank all items for each of the five category types, with no ties allowed.

Tables 2 through 4 from the Survey paper [6] show the consensus priorities for each of the first three category types as determined by a traditionally weighted scoring technique called the Borda count. [10] The Survey paper provides complete details of the method and results of the statistical analyses. Applying this procedure to the Survey category types, the following Borda score-weights were assigned: the first-placed items (highest priority or most significant) on every ballot receive scores of *n_i_*, the second-placed items receive scores of *n_i_* − 1, and so forth, until the lowest priority or least significant items on the ballot receive scores of 1. Scores were assigned to each of the 459 ballots from the respondents individually and then summed over all ballots within the category type of interest.

Items were ranked in descending order by the Borda score; i.e., the highest score is the “winner.” In short, the Borda score is a weighted mean with a particular assignment of weights to ballot positions. These Borda count orderings are referred to throughout the paper as the “global consensus” orderings. The global consensus order may not be the same as the order when only rank 1 votes are considered. For example, *Fabrication Tools* in Table 4 received 109 rank 1 votes, 61 rank 2 votes, …, and 44 rank 8 votes. All of the remaining 7 items in Table 4 received fewer than 109 rank 1 votes. The median rank of the underlying random variable was estimated to be 3 ±0.29. The global consensus is that *Fabrication Tools* is second to *Sensors* as a priority activity. Appendix B of the Survey paper [6] contains the definition for the 95 % confidence interval (CI) and describes the methodology in detail.

## 3. Assigning Priorities to USMS Measurement Needs for Nano-Electrotechnologies

As noted earlier, there is a large number of measurement needs for nano-electrotechnologies and limited resources to address them (both domestically and globally). Consequently, there is a need to rank these in priority order so that resources can be applied to addressing those needs that are most important to accelerating innovation.

For this analysis we assumed that the set of 64 MNs identified in the 2006 USMS assessment may serve as a proxy for the universe of nano-electrotechnology measurement needs. We then apply a process to analyze and rank this set in priority order using the taxonomy developed for the 2008 NIST-Energetics-IEC TC 113 Survey on Nano-Electrotechnologies. [6] As a proof-of-concept, we use the following method for assigning priorities to the 64 nano-electrotechnology USMS case studies of MNs listed in Appendix A.

### 3.1 Tagging Methodology for USMS Measurement Needs

A “tagging” process was employed to provide a consistent set of information from each MN. The tags correspond to the first three category types and ranked items given in Table 1, namely Properties, Products, and Cross-Cutting Technologies. Through this process we were able to uniformly gather a set of priority information for each MN that corresponded to the same ranking choices given to the Survey respondents.

A set of “items-tags” (i.e., ranked items from the Properties, Products, and Cross-Cutting Technologies category types) was selected for each of the 64 USMS MN case studies. Selection of tags was based on best scientific and engineering judgment, given the information presented in the case studies. Only one ranked item was assigned from each Survey category type to each USMS MN. Examples of the actual tags assigned to case studies for this analysis are shown in Table 5. For this proof-of-concept study, only three respondents participated in the tagging process.

### 3.2 Tagging Results

**Fig. 1:**
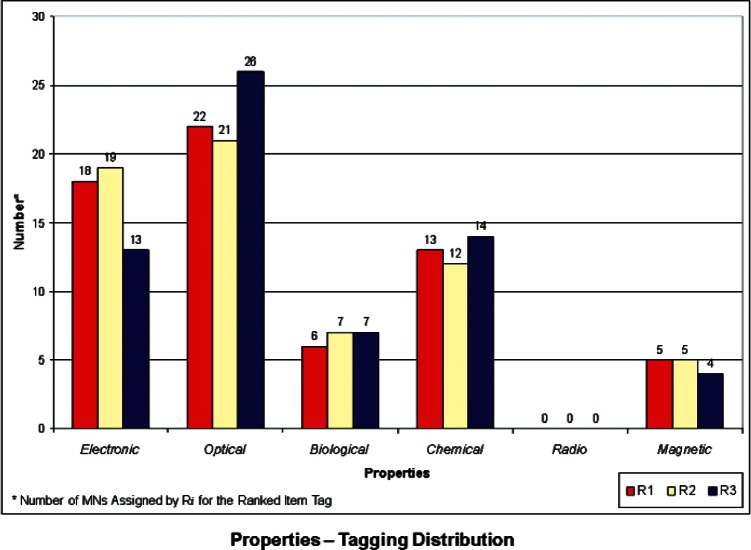
Tag Distribution for Properties.

We developed a set of histograms to illustrate the distributions of Properties, Products, and Cross-Cutting Technologies ranked “items-tags” that the 3 respondents assigned to each of the 64 USMS MN case studies. These are shown in Figs. 1 through 3. These histograms give rise to some general observations and also provide a basis for comparison with the NIST-Energetics-IEC TC 113 Survey, as outlined below.

**Fig. 2:**
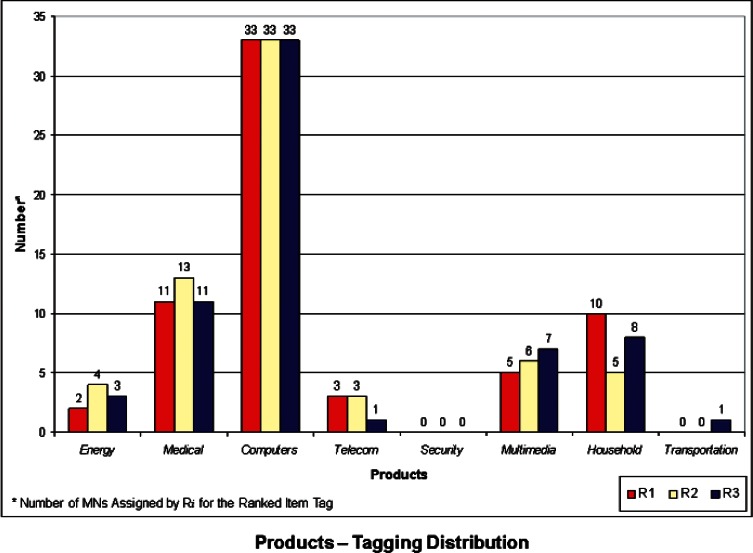
Tag Distribution for Products.

**Fig. 3:**
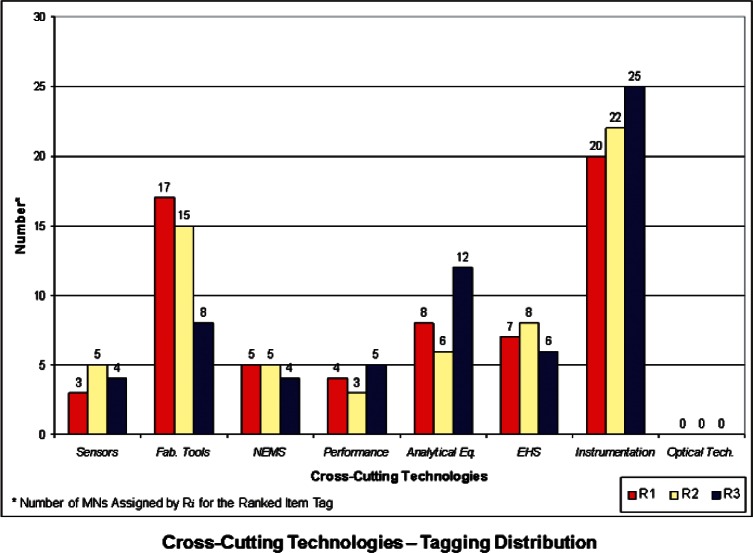
Tag Distribution for Cross-Cutting Technologies.

**Fig. 4:**
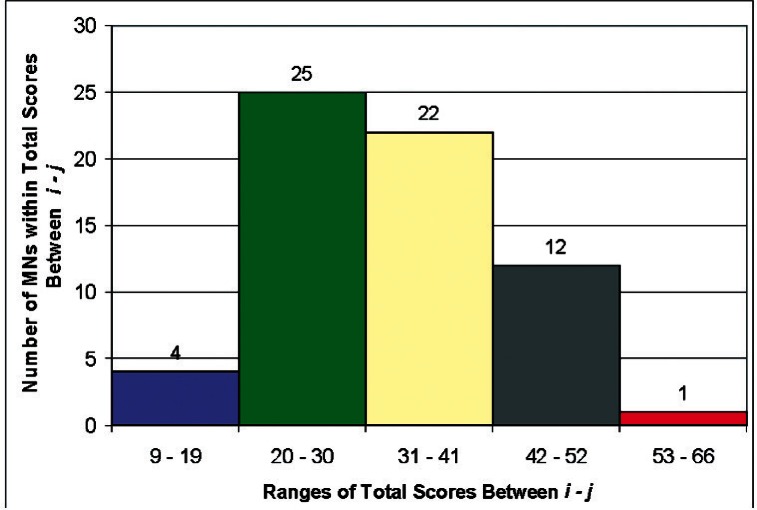
Distribution of Total Scores for the 2006 USMS MNs in Nano-electrotechnologies.

- **Properties** (Fig. 1) – The properties most frequently identified in the USMS MN cases studies fall within the areas of *Optical* and *Electronic*. This is somewhat comparable to the priorities identified in the Survey (refer to Figs. 4 and 9 in [6]). However, the overwhelming priority from the Survey was *Electronic* properties, compared with the USMS MN case studies, for which *Electronic* and *Optical* properties share large portions in the distribution of tags for properties. In the Survey, *Optical* properties received low priority rankings. *Biological* properties have a moderate but similar share in both the USMS MN tagging and the Survey.
- **Products** (Fig. 2) – The Product category most frequently identified in the USMS MN case studies was *Computers*, followed (but not closely) by *Medical* products. However, the product categories of *Computers*, *Medical*, and *Energy* in the Survey itself (refer to Figs. 5 and 10 in [6]) all have relatively high priority rankings. A possible reason for this is the more recent emphasis worldwide on energy as a priority compared to when the 2006 USMS report was conducted. While important, energy was not viewed to be at such a critical juncture in 2006 as it is today. In the medical field, recent rapid advances in innovative fields may be fueling a greater need for standards. *Telecommunications* received a relatively large share of high priority rankings, compared with the USMS MN case studies, where it only appeared in a few cases.
- **Cross-Cutting Technologies** (Fig. 3) – In this category, *Instrumentation* has the highest number of assignments, followed by *Fabrication Tools* and *Analytical Equipment*. When compared with the Survey (refer to Figs. 6 and 11 in [6]), only *Fabrication Tools* follows the same pattern. In the Survey, *Instrumentation* actually was ranked at the lowest priority. *Sensors* was highest in priority among the eight items for Cross-Cutting Technologies in the Survey, but very seldom assigned as a tag for the USMS MN case studies. In some respects, this may be an artifact of attempting to match the Survey categories to the USMS MN case studies; the latter of which were developed with a different perspective and not for the taxonomy used in the Survey. This is particularly true where overlap may exist between *Instrumentation* for testing and controlling fabrication processes or *Analytical Equipment* for measuring electro-technical properties. It may be difficult to distinguish which category is more appropriate in some cases. This sort of anomaly might be removed if the case studies were written more clearly or with these particular categories in mind. Another anomaly is Cross-Cutting Technology Item *Environmental Health and Safety (EHS)*, which was identified as a relatively high priority in the Survey, but was identified in only a small number of the USMS MN case studies. This is another case of a change in global priorities and interests—with the advent of more nanotechnologies into the marketplace and on the drawing board, interest in their safety and impact on the environment has increased.

We also compared the distributions of assigned USMS MN tags for Properties, Products, and Cross-Cutting Technologies with the high priority rankings from the Survey. Table 6 illustrates the results of this analysis.

The scorings in the column on the right in Table 6 show the total score for each MN based on the Survey Borda global consensus ranks. This total score is the sum of the Borda ranks for each of the three ranked Survey items which the respondents assigned to an MN. That is, each MN has three tags, one each from Properties, Products, and Cross-cutting Technologies. The minimum total score that the three respondents could assign a given MN is 3(1+1+1) = 9 (highest priority). The maximum total score that they could assign is 3(6+8+8) = 66 (lowest priority). Thus, a low score in the far right column indicates that this USMS MN has a very high priority based on the Survey’s global consensus. Alternatively, a high score indicates that this USMS MN has a very low priority based on the Survey’s global consensus. For example, the lowest score of 15 received for the MN “Carbon Nanotube Materials” indicates that the tagging for Properties, Products, and Cross-Cutting Technologies was highly correlated to high rankings in the original Survey. The high score of 54 received for MN “Self Assembly of Soft Nanomaterials” indicates that tagging results for this MN were the least correlated with high-ranked items in the original Survey.

The standard deviation for the total scores given in Table 6 is ±9. Figure 4 shows the distributions for the number of MNs receiving totals scores in the bands with total score widths of 10. The first bar is the number of MNs with scores between 9 and 19; the second bar is the number of MNs with scores between 20 and 30; and so on, with the fifth bar having scores between 53 and 66. Figure 4 suggests that, using the tagging process, the Survey respondents would assign 4 MNs the highest priority and 1 MN the lowest priority. They would assign 25 MNs high priority and 12 MNs low priority. The middle band has 22 MNs, indicating that those MNs are neither high nor low priority. Because the MNs are distributed among all 5 bands, we conclude that the set of 64 MNs correlates with the priorities of the Survey respondents.

## 4. Conclusions

We have successfully demonstrated as a viable proof-of-concept a method for placing in priority order USMS MNs by assigning ranked items from an independent survey to the USMS MNs. Our analyses suggest that from the perspectives of the 459 Survey respondents the priority ranking of the 64 USMS MN case studies for nano-electrotechnologies given in Appendix A is consistent with the Survey’s global consensus rankings. That is, by comparing the tagging selections with those of the Borda global consensus rankings in the Survey, we have established that the ranked set of 64 MNs correlates with the priorities of the Survey respondents.

Three additional considerations are in order. First, the case studies used in the 2006 USMS assessment were not written with the Survey tagging concept in mind. As a result, interpretation of the case studies to identify the most appropriate tags may be in some cases relatively subjective. Second, as might be expected, global priorities for business, energy, medicine, environment, and other areas have changed since 2006. This change in global priorities may be reflected in a few cases by differences between the ranked items in Survey taxonomy categories and the content of some USMS MN case studies. Third, the USMS MN case studies were written by a narrow segment of the U.S. measurement community, whereas the Survey was developed and responded to by a much broader international measurement community. Even with these considerations, the process was still able to provide a viable proof-of-concept.

## 5. Website For Downloading the 64 USMS MNs on Nano-electrotechnologies

The one-page summaries of the 64 nano-electrotechnology case studies are a subset of the measurement needs given in Appendix B of the June 2006 USMS Assessment Report [7]. The Website for downloading the 64 nano-electrotechnology case studies is http://nvl.nist.gov/pub/nistpubs/jres/114/4/Appendix-A-for-NIST-Energetics-USMS-MN-Priorities-06Jul09.pdf

## Figures and Tables

**Table 1:** Category Types and Rank Items Employed in the Survey [denotes abbreviation in data tables]

1. Properties
• Electronic and Electrical [Electronic]
• Optical [Optical]
• Biological [Biological]
• Chemical [Chemical]
• Radio Frequency [Radio]
• Magnetic [Magnetic]
2. Products
• Energy (production, conversion, and storage) [Energy]
• Medical Products [Medical]
• Computers (PDA and similar, laptop, desktop, mainframe) and Computer Peripherals (printers, monitors/displays, etc.) [Computers]
• Telecommunication and Data Communications (wireless and wired-physical connection) [Telecom]
• Security and Emergency Response Devices and Applications [Security]
• Multimedia Consumer Electronics [Multimedia]
• Household and Consumer Applications [Household]
• Transportation (sea/water, ground, air, space) [Transportation]
3. Cross-Cutting Technologies
• Sensors (chemical, physical, mechanical, etc.) [Sensors]
• Fabrication tools for integrated circuits (electronic, photonic, optoelectronic, and mechanical) [Fab. Tools]
• Nano-Electromechanical systems [NEMS]
• Performance and reliability assessment for nanoelectronics [Performance]
• Analytical equipment and techniques for measurements of electro-technical properties [Analytic Eq.]
• Environment, Health, and Safety (EHS) applications and effects [EHS]
• Instrumentation (test equipment and industrial process control for use in fabrication) [Instrumentation]
• Optical technologies (optoelectronics and illumination) [Optical Tech.]
4. General Discipline Areas
• Measurement and Performance [Measurement]
• Design and Development [Design]
• Health, Safety, and Environment (HSE) [HSE]
• Dependability and Reliability [Dependability]
• Electromagnetic Compatibility [Compatibility]
• Terminology, Nomenclature, and Symbols [Terminology]
5. Stages of Economic Model
• Basic Technical Research [Research]
• Technology Development (prototype development) [Development]
• Initial deployment [Deployment]
• Commercialization (large-scale, high-volume manufacturing) [Commercialization]
• End of initial use by the Customers-Consumers (End of Initial Usefulness) [End-of-Usefulness]
• End-of-Life (disposing and recycling) [End-of-Life]

**Table 2:** Consensus Priority Rankings for Properties [6]

	Raw Data						Median and 95 % Cl	Borda Score	Borda Global Consensus Rank
	Rank 1	Rank 2	Rank 3	Rank 4	Rank 5	Rank 6			
Electronic	292	57	58	26	13	13	1(+0.07)	2,386	1
Optical	17	115	112	105	78	32	3(±0.15)	1,628	2
Biological	68	73	68	75	77	98	4(±0.22)	1,522	3
Chemical	37	86	70	68	113	85	4(±0.22)	1,447	4
Radio	34	83	69	78	63	132	4(±0.29)	1,387	5
Magnetic	11	45	82	107	115	99	4(±0.15)	1,269	6

**Table 3:** Consensus Priority Rankings for Products [6]

	Raw Data								Median and 95 % Cl	Borda Score	Borda Global Consensus Rank
	Rank 1	Rank 2	Rank 3	Rank 4	Rank 5	Rank 6	Rank 7	Rank 8			
Energy	130	94	69	52	34	37	18	25	3(±0.22)	2,680	1
Medical	85	103	85	57	41	45	26	17	3(±0.22)	2,564	2
Computers	109	63	60	59	57	52	31	28	3(±0.22)	2,442	3
Telecom	57	82	72	89	72	43	29	15	4(±0.22)	2,397	4
Security	25	43	62	67	75	77	51	59	5(±0.22)	1,900	5
Multimedia	22	39	47	59	72	65	83	72	5(±0.22)	1,747	6
Household	20	12	39	30	47	76	119	116	7(±0.22)	1,398	7
Transportation	11	23	25	46	61	64	102	127	6(±0.22)	1,396	8

**Table 4:** Consensus Priority Rankings for Cross-Cutting Technologies [6]

	Raw Data								Median and 95 % Cl	Borda Score	Borda Global Consensus Rank
	Rank 1	Rank 2	Rank 3	Rank 4	Rank 5	Rank 6	Rank 7	Rank 8			
Sensors	100	94	60	49	51	45	34	26	3(±0.22)	2,496	1
Fab. Tools	109	61	66	52	47	40	40	44	3(±0.29)	2,387	2
NEMS	59	71	59	58	65	45	46	56	4(±0.29)	2,156	3
Performance	55	54	58	57	57	61	60	57	5(±0.29)	2,039	4
Analytical Eq.	30	57	54	70	80	74	58	36	5(±0.22)	2,007	5
EHS	71	40	45	39	48	54	66	96	5(±0.29)	1,895	6
Instrumentation	13	39	58	73	60	71	84	61	5(±0.22)	1,772	7
Optical Tech.	22	43	59	61	51	69	71	83	5(±0.29)	1,772	7

**Table 5:** Selected Examples of Tagging for USMS MN Case Studies

USMS MN Case Study Title	Property Tag	Product Tag	Cross-cutting Technology Tag
Nanomagnetic MRI Contrast Agents (p. A-52)	*Optical*	*Medical*	*EHS Application and Effects*
Cell-Based Analysis Using Lab-on-a-Chip Technologies (p. A-12)	*Electronic and Electrical*	*Medical*	*Fabrication Tools*
Nanoscale Chemical Characterization of Advanced Materials (p. A-63)	*Optical*	*Computers*	*Fabrication Tools*

**Table 6:** Priorities for USMS Nano-Electrotechnology Measurement Needs by Total Score. Based on Borda Global Consensus Rank. The lower the total score the higher the priority.

Appendix A: Page No.	Measurement Need (MN) Title	TOTAL SCORE Based on Borda Global Consensus Rank
A-1	Carbon Nanotube Materials	15
A-2	Quantum Computing	18
A-3	Nanoscale Integrated Circuits: Dimensional Control	18
A-4	Advanced CMOS Gate Stacks for Next Generation Integrated Circuit Devices	18
A-5	Top Down Micro/Nano Manufacturing	20
A-6	Nanostructured Materials for Photovoltaic	21
A-7	High Accuracy Dimensional Metrology for Manufacturing	21
A-8	Integrated Circuit Overlay Metrology	21
A-9	Sub-50 nm Lithography	21
A-10	Small Particle Monitoring For Advanced Semiconductor Manufacturing	21
A-11	Advanced DNA Analyses Using Lab-on-a-Chip Technology	22
A-12	Cell-Based Analysis Using Lab-on-a-Chip Technologies	22
A-13	Sub-10 nm SEM Metrology Tools	23
A-14	Nanoscale Biological Imaging	24
A-15	Compound Semiconductor Cluster Tools	25
A-16	Molecule-Based Nanoelectronics	26
A-17	Nanomanufactured Components	26
A-18	Multi-Layer Nanostructures for Electronic and Photonic Devices	26
A-19	In-line Inspection and Factory Control Equipment	26
A-20	Interfacial Characterization Instrumentation	26
A-21	Semiconductor Industry Defect Metrology Tools	26
A-22	Nanoimprint Lithography (NIL)	27
A-23	Nanocrystal Biophotonic Sensors	27
A-24	Current Flow in Nanoscale Electronic Devices	27
A-25	Next Generation Electrical Instrumentation	29
A-26	Next-Generation Active Nanodevices	30
A-27	Single Molecule Optical Measurement	30
A-28	Integrated Circuit Optical Linewidth Metrology	30
A-29	Single Biomolecule Detection, Classification, and Measurement	30
A-30	Integrated Circuit Photomask Metrology	31
A-31	Nanomanufacturing	32
A-32	Sidewall Characterization Instrumentation	32
A-33	Health Care/Nanotechnology - Cancer Diagnosis and Treatment	33
A-34	Advanced Force Measurements in Nanotechnology	33
A-35	Advancing the Fundamental Science of Nanobiotechnological Systems	33
A-36	In-line/Real-time Analytic Tools for Measuring Sub-10 nm Defects	34
A-37	Dopant Distribution Instrumentation	34
A-38	Atomic Mapping Instrumentation	34
A-39	MEMS	34
A-40	Dimensionally Critical Nanomanufacturing	35
A-41	Airborne Contamination in Semiconductor Wafer Processing	36
A-42	Carbon Nanotube Identification	36
A-43	Multilayer Film Structures for Electronics and Optics Industries	36
A-44	Novel Materials for Nanoscale Diffusion Barriers in Microelectronics	36
A-45	Next-Generation Optical Microscopes	36
A-46	Nano-Scale Drug Delivery	38
A-47	Directed Nanoscale Assembly	39
A-48	Strained-Layer Engineering for High Performance Electronic and Optoelectronic Devices	39
A-49	Instrumentation for Measurement of Electrical Properties at the Nanoscale	39
A-50	SEM and AFM Modeling for Semiconductor Electronics and Nanotechnology	40
A-51	Micro/Nano-Technology	40
A-52	Nanomagnetic MRI Contrast Agents	42
A-53	Atomic-Precision Imaging to Aid Development of New Materials	42
A-54	Hard Disk Stack Metrology	43
A-55	Carbon Nanotubes (CNTs)	44
A-56	Toxicology of Nano-Particles in Biological Systems	44
A-57	Next Generation Electrotechnical Products, Components, and Raw Materials	45
A-58	Magnetic Data Storage	45
A-59	Nanomanufactured Components in Complex Fluids	46
A-60	Spin Metrology Tools	47
A-61	Hard Disk Sheet Magnetoresistive	48
A-62	Scanning Electron Microscope Nanocharacterization	49
A-63	Nanoscale Chemical Characterization of Advanced Materials	51
A-64	Self Assembly of Soft Nanomaterials	54
